# A Knitted Sensing Glove for Human Hand Postures Pattern Recognition

**DOI:** 10.3390/s21041364

**Published:** 2021-02-15

**Authors:** Seulah Lee, Yuna Choi, Minchang Sung, Jihyun Bae, Youngjin Choi

**Affiliations:** 1 Department of Electrical and Electronic Engineering, Hanyang University, Ansan 15588, Korea; seulah@hanyang.ac.kr (S.L.); chldbsk2220@hanyang.ac.kr (Y.C.); wdrac331@hanyang.ac.kr (M.S.); 2 Human-Tech Convergence Program, Department of Clothing and Textiles, Hanyang University, Seoul 04763, Korea

**Keywords:** knitted sensor, wearable strain sensor, data glove, fabric sensor, pattern recognition

## Abstract

In recent years, flexible sensors for data gloves have been developed that aim to achieve excellent wearability, but they are associated with difficulties due to the complicated manufacturing and embedding into the glove. This study proposes a knitted glove integrated with strain sensors for pattern recognition of hand postures. The proposed sensing glove is fabricated at all once by a knitting technique without sewing and bonding, which is composed of strain sensors knitted with conductive yarn and a glove body with non-conductive yarn. To verify the performance of the developed glove, electrical resistance variations were measured according to the flexed angle and speed. These data showed different values depending on the speed or angle of movements. We carried out experiments on hand postures pattern recognition for the practicability verification of the knitted sensing glove. For this purpose, 10 able-bodied subjects participated in the recognition experiments on 10 target hand postures. The average classification accuracy of 10 subjects reached 94.17% when their own data were used. The accuracy of up to 97.1% was achieved in the case of grasp posture among 10 target postures. When all mixed data from 10 subjects were utilized for pattern recognition, the average classification expressed by the confusion matrix arrived at 89.5%. Therefore, the comprehensive experimental results demonstrated the effectiveness of the knitted sensing gloves. In addition, it is expected to reduce the cost through a simple manufacturing process of the knitted sensing glove.

## 1. Introduction

With the advancement of recent human-machine interaction (HMI) technology, a variety of human-device interfaces have been proposed in various fields such as the rehabilitation, sports, and robot industry [1,2,3,4,5,6]. In particular, the data glove is a representative HMI device, which can be used as a measurement device for rehabilitation training or as a tool for robotic teleoperation in dangerous working environments [7,8]. The human hand’s multiple degrees-of-freedom (DoFs) is one of the most critical factors on the body for daily activity. The data glove can be classified as a sort of wearable sensor, which has flexibility and conductivity. The flexible wearable sensors, in contrast with hard and heavy materials like metals, accelerate the development of HMI. For this purpose, the data glove based on electronic textiles (e-textiles) has attracted considerable interest in the development of advanced wearable devices. Also, the data glove mainly makes use of strain sensors, which require flexibility and wearability. Conventional strain sensors were attached to the glove by gluing, sewing, printing, and taping [9,10,11]. On the other hand, inertial measurement units (IMUs) have been attached to the glove [12,13], but they made the glove uncomfortable, rigid, and bulky because the processing board and wires should be attached to specific areas such as the palm, finger, and backside of the hand. As an alternative, flexible wearable sensors based on e-textiles are a good choice for hand motion monitoring and recognition, because they are comfortable to wear and also they do not influence finger and palm movements.

Recent studies on sensing gloves include rehabilitation training systems [14], hand motion capturing systems [15], tracking systems [16], and novel glove designs [17,18,19,20], e.g., Ryu et al. [18] proposed a knitted sensing glove system, which used a conductive yarn by a knitting machine and the results of the strain data showed stability and linearity. However, the sensors enclosed only the proximal interphalangeal (PIP) joint of the index and middle fingers in a circular way, and could also be uncomfortable to wear because they had seams (called sewing line). Han et al. [20] introduced the knitted flexible sensor based on the loop structure. The knitted glove with strain sensor was able to monitor the hand motions. However, the amount of electrical resistance change was small due to low conductivity, and thus there were limitations to monitor the entire movements because the sensor was knitted into only one finger. Nishiyama et al. [21] developed a wearable sensing glove using hetero-core fiber-optic nerve sensors. Although this glove resulted in good stability and repeatability, the process of manufacturing sensors was complicated and required attaching sensors into gloves separately. Ayodele et al. [22] developed a weft knit smart data glove, but it was insufficient for pattern recognition evaluation to verify the performance of the proposed glove. Some studies [11,13,23] have shown several examples that the strain sensor could be fabricated using conductive materials and attached to a commercial glove. 

The aforementioned studies required a lot of time and effort to go through different processes such as foundation and sewing. In this paper, we propose an integrated data glove that can be arranged at once by inserting a sensor into each finger. In the past, we had tried to find appropriate materials for the proposed glove [24]. This paper describes a novel knitted sensing glove with high-sensitivity, excellent durability, that is inexpensive, flexible, and has a simple manufacturing process. In addition, experimental studies are conducted to systematically investigate the performance of the developed sensing glove and its hand motions pattern recognition. The developed glove fabricates as a seamless type for the comfort of wearing, and it is composed of strain sensors knitted with conductive yarn and a glove body with non-conductive yarn. Thus, the knitted sensing glove can measure strains when the fingers and wrist are flexed and extended. To verify the performance of the knitted sensing glove, the changes of the electrical resistances of the proposed data glove are investigated according to the movements of finger and wrist. Additionally, to confirm the practicality of the knitted sensing glove, a convolutional neural network (CNN) is applied to the acquired data set for the hand postures classification. Consequently, clothing materials can be utilized so as to give a comfortable fit of the data glove at a low cost, and also it is expected that they can be extended to a human body monitoring system that is possible to wear in daily life. 

In order to emphasize the differences between the conventional data glove and the proposed glove, the well-known interactive glove [5] is adopted and compared with the proposed glove. The first difference between the interactive glove (Design Research Lab Berlin) and the proposed one would be how to fabricate it. The interactive glove is made of classical gloves cut and combines leather with knitwear, but the proposed sensing glove is fabricated at all once by knitting technique without sewing and bonding, which is composed of strain sensors knitted with conductive yarn and a glove body with non-conductive yarn. The second difference is that all parts of the glove have elasticity; therefore, there is no need to customize it for the size of the hand. The third difference is that the proposed glove can detect the movements of the thumb and wrist. Since the role of the thumb when the hand moves is very important, sensing the thumb is essential. Also, wrist motion detection can extend the number of realizable hand postures.

## 2. Materials and Fabrication

Since the data glove makes measurements of hand and finger motions, rapid response and good sensitivity are required. For these purposes, the developed sensing glove is knitted using the Shima Seiki whole garment machine (Shima Seiki©, Wakayama, Japan) based on SDS one apex apparel CAD (Shima Seiki©, Wakayama, Japan). Also, the whole garment technology as manufacturing without sewing stitches line provides comfort for wearers and saves time. The developed sensing glove is knitted with flexible and lightweight materials, which consist of non-conductive yarn and conductive yarn. Non-conductive yarn for a glove body is polyester and conductive yarn for the sensor is 99% silver-coated nylon (Statex© Shieldex®, Bremen, Germany, 117/17 dtex 2-ply), which has linear resistance of approximately 30 Ω/cm. The knitted structure is made of consecutive loops, which is more elastic than other fabrics such as woven and nonwoven fabrics, and also the spandex is arranged as a whole to give more elasticity.

**Figure 1:**
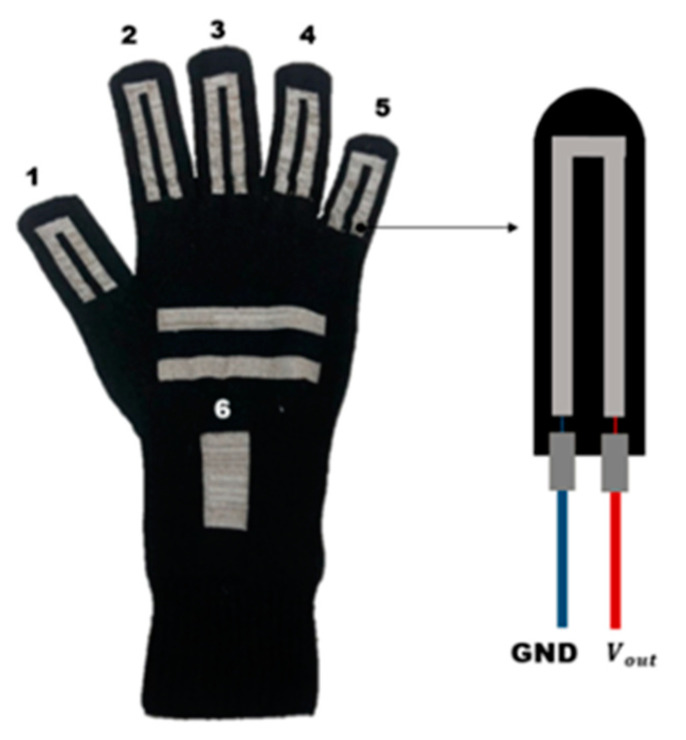
The strain sensor placements of the knitted sensing glove [24].

To reduce the signal noises of the knitted sensing glove, we adopted the method of knitting stitch as the intarsia knitting. The intarsia knitting can arrange conductive yarn only in the strain sensor area, to minimize interference with non-conductive regions. As shown in Figure 1, six strain sensors were knitted on the developed sensing glove along with the finger, thumb, and wrist directions. Five sensors were placed through the proximal interphalangeal (PIP) and metacarpophalangeal joints (MCP) of four fingers and thumb, and the remaining one was also placed on the wrist. In addition, the different sizes of the sensors were inserted in the glove according to the lengths of the fingers and the wrist, but they did not significantly affect the results for application use. For the extensive study and the comparisons between the materials to measure the strain, we have used the sensor design proposed in our preliminary study [24].

**Figure 2:**
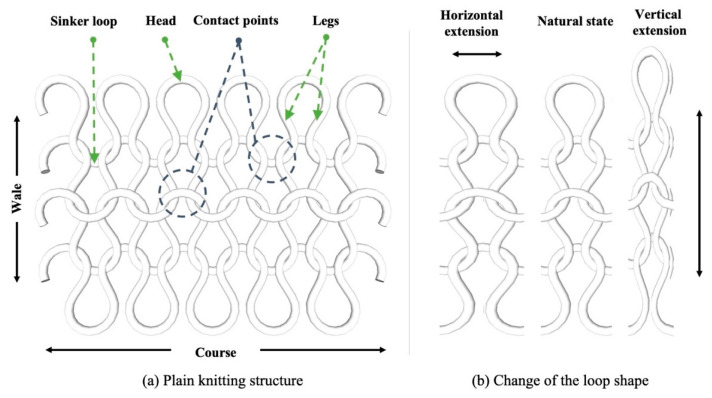
Schematic diagram of knitted sensing glove; (**a**) plain knitting structure; (**b**) changes of the loop shapes.

The knitting forms a series of single loops that contact adjacent parts such as the sinker loops, legs, and heads, as shown in Figure 2a. Conductive yarn as a strain sensor plays a significant role in the knit structure [25,26,27,28]. Since the knit has stretchability, the contact area decreases as the distance between adjacent loops increases in the conductive area as shown in Figure 2b which describes the nature of knits having the extensions of horizontal and vertical directions, explaining that the contact points and pressure may vary depending on the structure of the sensor. On the other hand, the contact pressure increases, and the electrical resistance on contact decreases, which can be described according to Holm’s theory [29].

(1) $$R_{c}\;=\;\frac{\rho }{2}\sqrt{\frac{\pi H}{nP}}$$

where ρ is an electrical resistivity of the fiber, *n* is the number of contact points, *P* is the contact pressure, and *H* is the fiber strength. While the electrical resistivity and strength of conductive yarn are constants, the number of contact points and contact pressure can be changed depending on the sensor’s structure and design. Therefore, the electrical resistance of the contact decreases when the contact pressure and the number of contact points increase inside the conductive knit [30]. 

## 3. Methods

### 3.1. Experimental Setup

All methods and procedures were approved by the Institutional Review Board(IRB) on Human Subjects Research and Ethics Committee at Hanyang University Hospital, Seoul, Korea (July 2020, HYUIRB-202007-011-1). A total of 10 able-bodied healthy subjects participated in this study. All subjects were informed of the experimental protocol before the experiments. They wore the knitted data glove on the right hand and the experiments were proceeded according to the researcher’s instructions.

In order to verify the performance of the developed sensing glove, three kinds of experiments were conducted regarding the knitted sensing glove. Firstly, we evaluated the characteristics of the knitted strain sensor. Secondly, the electro-mechanical properties of the finger movement were assessed by using the relative changes in electrical resistance to the angle and speed of the flexed and extended motions. Finally, the pattern recognition experiments regarding 10 target hand postures were demonstrated for practical verifications. The experiments were performed and analyzed using MATLAB R2018a (Mathworks, Natick, MA, USA), SPSS version 21 (IBM SPSS statistics 21, SPSS Inc., Chicago, IL, USA), Python, and Pytorch (Facebook Co., Menlo Park, CA, USA). 

### 3.2. Characterization of the Knitted Strain Sensor

**Figure 3:**
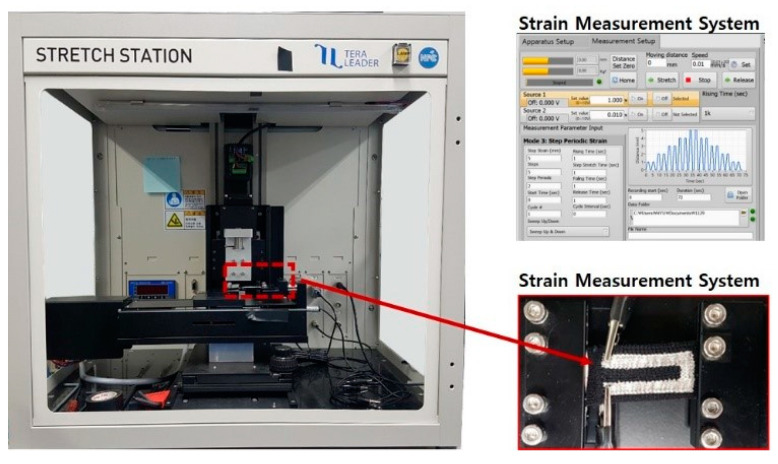
A universal strain testing machine for the knitted strain sensor.

The relative changes in electrical resistance while the knitted strain sensor being deformed were measured using a universal strain testing machine (Teraleader, Daejeon, Korea), as shown in Figure 3. The knitted strain sensor was evaluated based on three programmable modes: continuous changes up to 30% of strain, electrical resistance changes in small stepwise stretching, and change in electrical resistance when cyclic strains are applied. Prior to the external force applied to the sensor, the initial electrical resistance was recorded, the change in electrical resistance was measured as the force increased, and the electrical resistance was finally recorded after when the force was removed.

### 3.3. Performance of the Knitted Sensing Glove

The strain signal of the sensing glove was measured using the voltage divider circuit as follow:

(2) $$V_{out}\;=\;\frac{R_{2}}{R_{1}+R_{2}}\;\times \;V_{in}\;\to \;R_{2}\;=\;\frac{V_{out}}{V_{in}-V_{out}}\;\times \;R_{1}$$

where *R*_1_ is a constant reference resistance and *R*_2_ is variable electrical resistance of the knitted sensing glove to be measured as the strain sensor and finally, we can calculate the electrical resistance *R*_2_ of the knitted strain sensor from the measured output voltage *V_out_*, the input constant voltage *V_in_*, and the constant reference resistance *R*_1_. In detail, variable output voltage *V_out_* ranged 0~5 V was measured by using the analog-to-digital converter existing inside the Arduino micro, and then the electrical resistance *R*_2_ of the knitted sensing glove was calculated from the input voltage of *V_in_* = 5 V and the reference resistance of *R*_1_ = 1000 Ω.

### 3.4. Pattern Recognition for 10 Target Hand Postures

**Figure 4:**
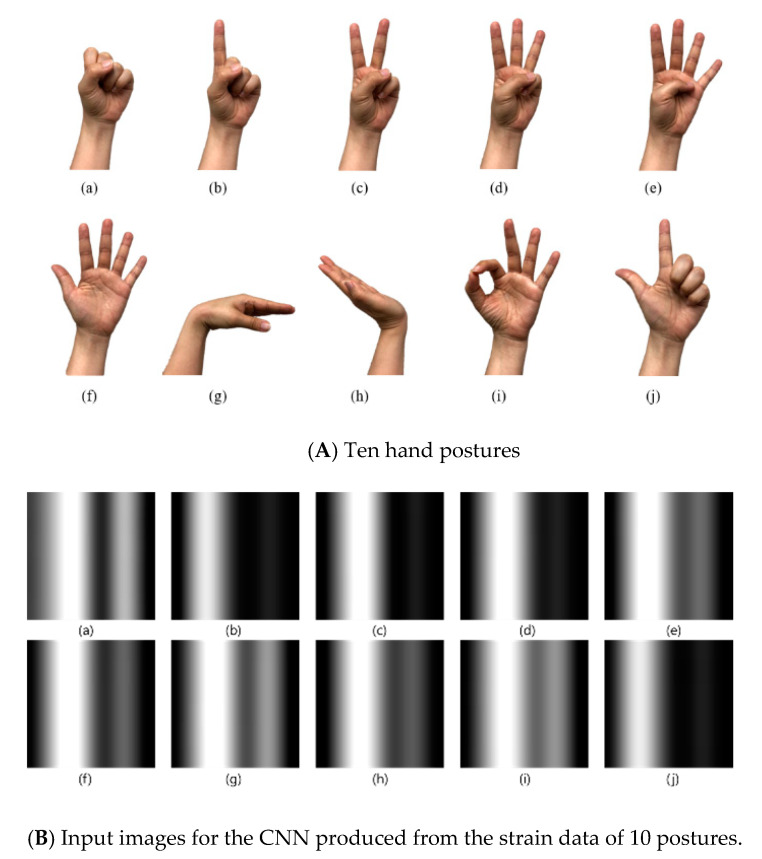
Ten hand postures and their images for the CNN (convolutional neural network) produced from the strain data for pattern recognition; where (**a**) grasp, (**b**) one, (**c**) two, (**d**) three, (**e**) four, (**f**) five (rest), (**g**) wrist flexion, (**h**) wrist extension, (**i**) okay, and (**j**) pinch.

**Figure 5:**
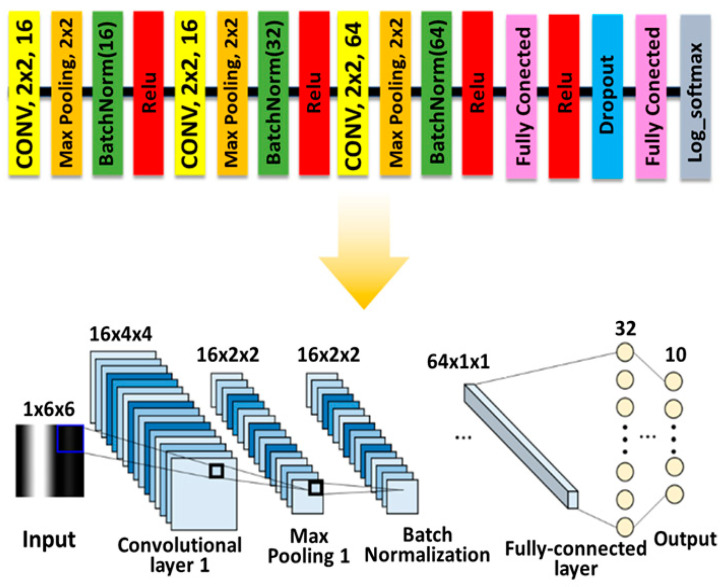
Architecture of the proposed convolutional neural network for hand postures classification.

The hand postures pattern recognition has been studied in various areas such as rehabilitation and robotics. To verify the feasibility of the developed knitted sensing glove, the strain data were collected from 10 different hand postures as shown in Figure 4A. For the data of the knitted sensing glove collected from 10 postures, a repeated-measures analysis of variance (RM-ANOVA) with Bonferroni was performed to assess statistical differences, where the level for significance was set at *p =* 0.001. The proposed network is composed of three convolutional networks and two fully connected layers, as shown in Figure 5. Each convolutional network consists of a 2 × 2 kernel convolutional layer, a max-pooling layer, and a batch normalization layer. The first fully connected layer does not have dropout, but the second fully connected layer has a dropout. The ReLU activation function is used at the end of each network layer and the log softmax function is used at the end of proposed network to produce the output with probability. The input of the network is a 6 × 6 image made with six samples taken on six channels as shown in Figure 4B. These input images captured from hand postures pass through each network layer and are converted into 10 posture classes with probability. 

## 4. Results

### 4.1. Characterization of the Knitted Strain Sensor

**Figure 6:**
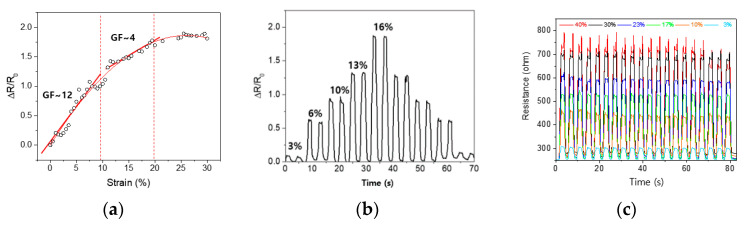
Characterization of the knitted strain sensor, where (**a**) continuous changes up to 30% of strain, (**b**) electrical resistance changes in the stepwise (3%, 6%, 10%, 13%, 16%, 13%, 10%, 6%, 3%) stretching, (**c**) the electrical resistance when cyclic strains are applied at a level of 3%–40% for 20 cycles.

Figure 6 shows the characteristics of the knitted strain sensor in three different modes such as continuous changes up to 30% of strain, electrical resistance changes in small stepwise stretching, and change in electrical resistance when cyclic strains are applied. The sensor is stretched to the range of 30% at a speed of 50 mm/min and the relative resistance change of the sensor is measured continuously as shown in Figure 6a. It demonstrates the linear response up to 10% strain (R^2^ = 0.97) with a strain gauge factor (GF) around GF = 12. According to the increase of strain, it is observed that the sensitivity is decreased and finally saturated. Figure 6b shows the dynamic performance with a repetitive stepwise stretch-release cycle, which was evaluated at the strain ranges of 3–16% with a constant frequency of about 0.25 Hz. Finally, the response of the knitted strain sensor with a wide range of the strains of 3% thru 40% for 20 cycles at 0.25 Hz is shown in Figure 6c. It shows good stability in overall strain ranges except for some extent of fluctuation at 40% of strain. 

### 4.2. Performance of the Knitted Sensing Glove

**Figure 7:**
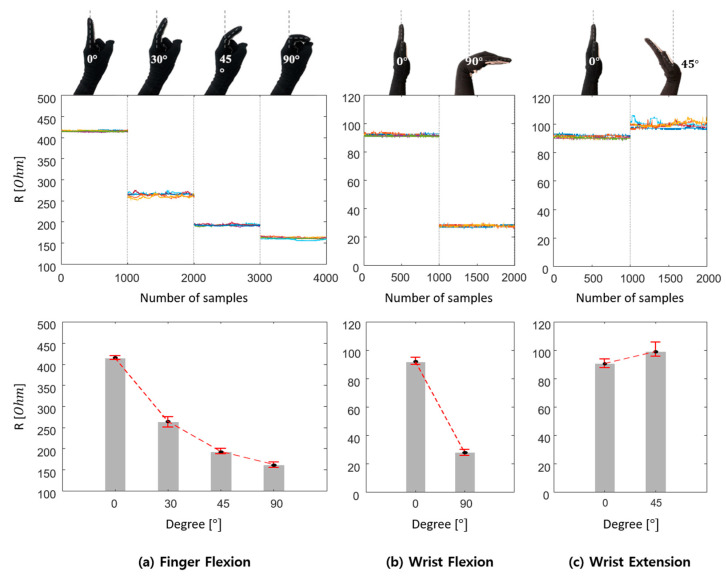
Strain characterization results of the knitted sensing glove: (**a**) index finger flexion; (**b**) wrist flexion; (**c**) wrist extension.

To verify the performance of the knitted sensing glove, both movements of index finger and wrist were measured by calculating the electrical resistance *R_2_* with a 1000 Hz sampling rate. First, the resistance changes were recorded according to the index finger flexion by five times as shown in Figure 7a. As the finger starts to be flexed, the distance between the knitted loops is closer, and the number of contact points of the sensor increases, and thus the electrical resistance decreases. Also, we could confirm that it becomes relatively hard to distinguish the finger flexion angles above 45° when being compared to the flexion angles below 45° because the GF (strain gauge factor) becomes small according to the strain increases. Another experiment was additionally conducted regarding flexion and extension of the wrist by five times as shown in Figure 7b,c. The electrical resistance values were maintained at about 92 Ω before flexion and extension of the wrist. The electrical resistance was greatly lowered when the wrist flexion was performed, and it increased a little bit when the wrist extension was made. This is because the electrical resistance depends on the increase or the decrease of contact points within the knitted sensor. Also, we were able to determine that the resistance change of the finger flexion was greater than those of the wrist flexion. 

**Figure 8:**
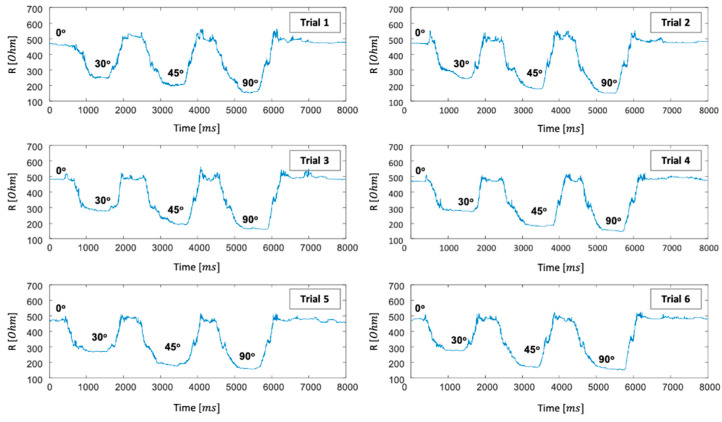
Electrical resistance variations according to the amplitudes of the index finger movements.

In detail, raw experimental data regarding the index finger (continuous flexion and extension) movements were suggested according to time sequences as shown in Figure 8, in order to know the electrical resistance variations according to the amplitudes of finger motions. The subject wearing the knitted sensing glove made the continuous index finger movements from 0° to 0°, via 30°, 45°, and 90°. It was tried six times, as shown in Figure 8. Although the electrical resistance values varied a little bit according to the different trials, the time series data show a similar pattern. The resistance values according to the flexion angles are enough to distinguish the amount of the flexion angles.

**Figure 9:**
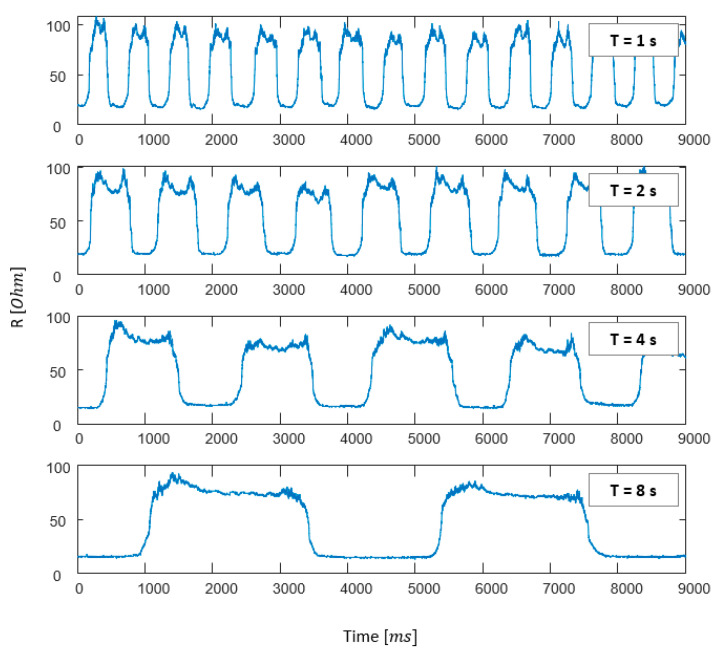
Repeated measurement results of the wrist movement with different speed conditions, where top graph shows 2 Hz (fast) flexion/extension, second 1 Hz, third 0.5 Hz, and bottom 0.25 Hz (slow), and *ms* stands for milliseconds.

Additionally, the experiment was conducted that the subject repetitively flexed and extended the wrist with different speeds such as 2 Hz (fast), 1 Hz, 0.5 Hz, and 0.25 Hz (slow) under the same conditions. As a result of the repeated measurements, there were little resistance differences in the flexion peak values as shown in Figure 9. Hence, the electrical resistance change of the developed sensing glove was little influenced by the wrist movement speed. However, it is possible that higher speed leads to higher resistance according to the degree of overlapping or folding of the sensor, which might be caused by the increased friction due to the characteristics of the fabric.

### 4.3. Pattern Recognition for 10 Target Hand Postures

**Figure 10:**
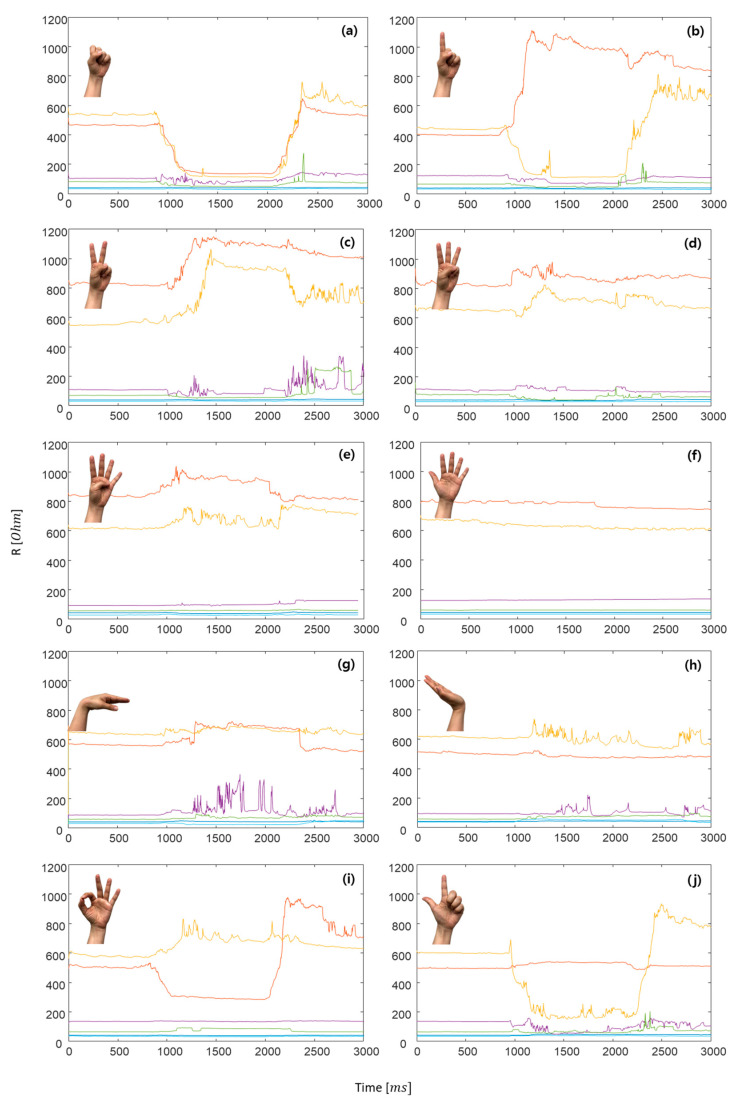
Electrical resistance variations according to 10 hand postures, where (**a**) grasp, (**b**) one, (**c**) two, (**d**) three, (**e**) four, (**f**) five (resting posture), (**g**) wrist flexion, (**h**) wrist extension, (**i**) okay, and (**j**) pinch, and *ms* stands for milliseconds.

**Figure 11:**
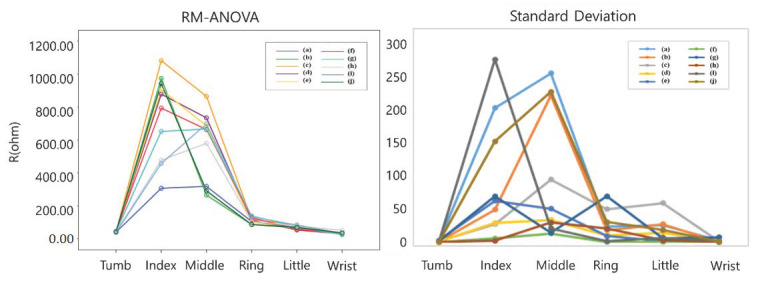
Repeated-measures analysis of variance (RM-ANOVA) and standard deviation of electrical resistances variations according to 10 hand postures, where (**a**) grasp, (**b**) one, (**c**) two, (**d**) three, (**e**) four, (**f**) five (resting), (**g**) wrist flexion, (**h**) wrist extension, (**i**) okay, and (**j**) pinch.

To confirm the feasibility of the knitted sensing glove, we conducted the pattern recognition of 10 hand postures after wearing the developed sensing glove on the hand. Strain signals (electrical resistance variations) of the knitted sensing glove were collected for 10 hand postures, as shown in Figure 10, where (a) grasp, (b) one, (c) two, (d) three, (e) four, (f) five (resting posture), (g) wrist flexion, (h) wrist extension, (i) okay, and (j) pinch. The electrical resistances decrease in the case of finger flexion, and they increase in the extension as well. In the grasping posture (a), the electrical resistances of all fingers, except the wrist, dramatically decreased until returning to rest posture. In the resting posture (f), the electrical resistances have almost not changed, and they have maintained almost constant values. For the wrist flexion (g) and extension (h), we could confirm the differences between their phases (rising and falling) as shown in Figure 10g,h, but their changes were around 10 Ω and relatively not big when being compared with those of the fingers. In addition, we could confirm the differences among six strain signals for 10 postures as shown in Figure 11, and the electrical resistance changes are clearly differentiated among the 10 postures. To further confirm their differences, we applied RM-ANOVA to the 10 postures. As a result, there were statistically significant differences among six strain signals for 10 postures (*p* < 0.001). Confirming the standard deviation, the electrical resistance changes of the index finger were most prevalent, and the resistance changes of the thumb were almost unchanged.

To further confirm the practicality of the developed sensing glove, the postures pattern classification of the hand and wrist was demonstrated wearing the sensing glove on the hand. The proposed CNN model was utilized for the hand postures classification, which was trained and validated using only the subject’s own data. Table 1 suggests the classification accuracy for 10 hand postures of 10 subjects. The average classification accuracy for all subjects was 94.17% when their own data were used. Of the 10 postures, grasping posture reached 97.1% with the highest classification accuracy. The resting posture indicates the lowest average accuracy of 90.7%, and the average accuracy for all postures was high over 90%. 

**Figure 12:**
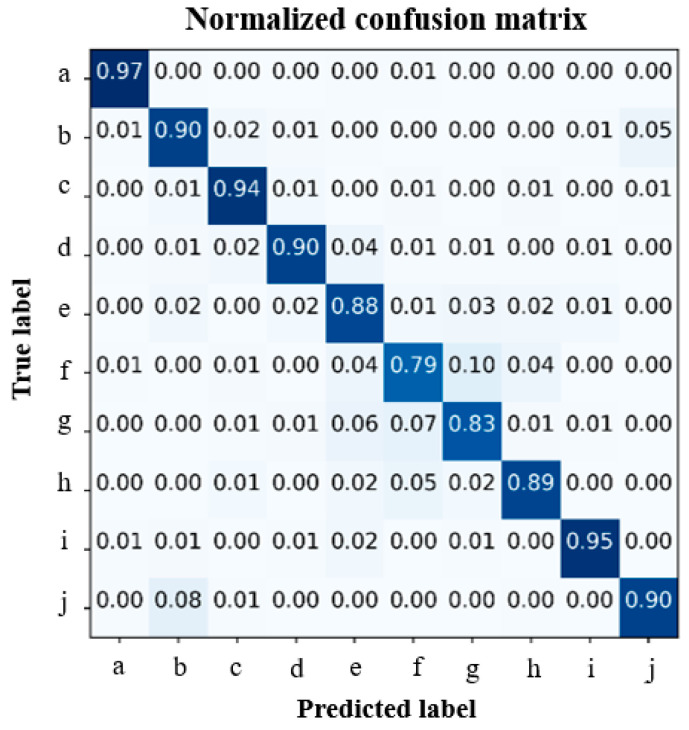
Classification accuracy expressed by confusion matrix obtained using all mixed data, where (**a**) grasp, (**b**) one, (**c**) two, (**d**) three, (**e**) four, (**f**) five (rest), (**g**) wrist flexion, (**h**) wrist extension, (**i**) okay, and (**j**) pinch.

In addition, the CNN model was trained and verified using all data mixed and collected from 10 subjects. Figure 12 shows the confusion matrix to express the classification accuracy for all mixed data. The average accuracy was 89.5%. All postures were above 83% except for the resting posture, especially, the resting posture was the lowest accuracy of 79%, and the grasping posture was the highest accuracy of 97%.

## 5. Discussion

In this study, we proposed a wearable knitted glove with strain sensors for pattern recognition. Four fingers, thumb, and wrist parts of the developed glove were knitted with strain sensors in a seamless way. For this purpose, whole garment technique was utilized to produce in one-step without cutting and sewing, and it was knitted by commercial clothing machine. Therefore, it is comfortable to wear because it is not only seamless, but also the sensor is integrally knitted into the glove. Additionally, it is expected that it can reduce the cost and process, ultimately for mass production.

In order to verify the performance of the developed sensing glove, we carried out several kinds of experiments. Firstly, the characteristics of the developed strain sensor were confirmed by three modes such as continuous changes up to 30% of strain, electrical resistance changes in small stepwise stretching, and change in electrical resistance when cyclic strains are applied. The electrical resistances proportionally responded to the amount of strain having good stability and repeatability. The basic fabric of sensor consists of a single jersey knit structure, and the deformation within the fabric structure might cause the drift in the response of sensor. Furthermore, it could be minimized through the optimization of the elastomeric yarn and knit structure. Secondly, the knitted sensing glove measures the electrical resistance changes in the wale direction that the flexion and extension of fingers and wrist make. The electrical resistances were gradually lowered when the flexion was performed. On the other hand, the electrical resistances became high when extension was performed. According to Holm’s theory, the number of contact points between the loops increases as the flexion is made, and thus it results in gradually lower electrical resistance. Thirdly, the number of contact points depends on the degree of flexion, so there was a difference in electrical resistance in accordance with the flexion angle. Thus, the developed sensing glove could monitor and be used for control in various fields such as wearable robotics, rehabilitation, and prosthesis. Finally, we measured the electrical resistances according to wrist flexion speeds. The electrical resistance change of the developed sensing glove was little influenced by the wrist movement speeds. 

After validating the performance of knitted sensing glove, we further demonstrated the applicability of the proposed glove to the hand postures classification. Prior to the classification, we confirmed the electrical resistance variations according to 10 different postures with the knitted sensing glove. The proposed glove is very efficient for evident patterns according to the hand postures. Consequently, we clearly confirmed different patterns for the 10 postures. As a matter of fact, the performance of the knitted strain sensor has somewhat individual differences depending on hand size, finger lengths, and movements. Thus, the classification result is slightly different for different individuals. The average classification was a high level of 94.17% when the subject’s own data were utilized to the CNN. In addition, the average classification accuracy was 89.5% when all data obtained from 10 subjects are mixed and used for the classification. Eight of 10 postures were recognized at approximately 90% or over 90%. Thus, the generalization might be acceptable. In detail, the accuracy of the resting posture was the lowest among 10 postures because there are many overlapping with other postures such as four fingers extension, wrist flexion, and extension. 

We should take note that the knitted sensing glove is different from previous studies on wearable sensing gloves from the viewpoint of manufacturing. Most of the previous studies on the wearable data glove did not consider the manufacturing process because it was not only bonded and sewn but also used materials that are not flexible and not fabric, such as electronic components and wire [8,9,10,11,12,13,15,21]. Since the hand and wrist have multiple DoFs, however, the data glove needs a flexible, lightweight, and embeddable sensors. In addition, it is noted that the previous studies have mostly focused on experiments limited to fabric evaluation as a part of glove [18,20,23]. These previous methods were insufficient to apply to the strain-type glove including fingers, thumb and wrist. However, it was verified that the knitted sensing glove proposed in the paper could be useful for hand postures classification in real-time. The knitted sensing glove might provide us an opportunity for commercialization in the areas of wearable health monitoring and robotic prosthesis since it can easily be worn by a variety of users.

## 6. Conclusions

This paper presents a knitted sensing glove for pattern recognition of hand postures. The performance of the knitted sensing glove was verified for various application fields such as wearable monitoring device, robotic prosthesis, robotic teleoperation, and so on. The paper dealt with sensor design, its fabrication and performance, and ultimately, hand posture pattern classification. The strain sensors of the proposed glove were made of silver-coated yarn as the conductive material. The sensing parts were inserted into the glove using the whole garment knitting technique for ease of commercialization. The developed glove would be useful to amputees as a device that allows them to rehabilitate using another hand, or control the myoelectric prosthesis. In particular, it is expected to be suitable for control of a multiple DoFs robotic hand because the data acquired from fingers and wrist could be utilized, respectively.

## Figures and Tables

**Table 1:** Classification accuracy of 10 hand postures using the proposed convolutional neural network (CNN) when their own data were used for the CNN.

	Subject										**Mean**
	1	2	3	4	5	6	7	8	9	10	
**(a) grasp**	97	99	97	95	97	97	99	96	97	97	97.1
**(b) one**	88	98	93	96	98	90	97	95	92	97	94.4
**(c) two**	95	98	94	90	93	96	94	97	95	97	94.9
**(d) three**	93	98	93	83	92	97	95	97	97	97	94.2
**(e) four**	93	97	95	90	95	95	94	92	90	96	93.7
**(f) five**	96	92	87	96	95	78	90	84	93	96	90.7
**(g)** **flexion**	93	88	87	96	97	93	84	91	93	93	91.5
**(h) extension**	94	98	90	96	94	84	93	94	97	94	93.4
**(i) okay**	96	97	96	97	96	96	97	98	97	98	96.8
**(j) pinch**	90	98	96	95	97	96	95	94	92	97	95
**Mean**	93.5	96.3	92.8	93.4	95.4	92.2	93.8	93.8	94.3	96.2	94.17

## Data Availability

Not applicable.
